# Canada geese (*Branta canadensis*) nesting on elevated structures in urban Indiana, USA

**DOI:** 10.1002/ece3.8735

**Published:** 2022-03-18

**Authors:** David J. Shearer, Timothy C. Carter, Benjamin J. O'Neal

**Affiliations:** ^1^ 5666 Department of Biology Ball State University Muncie Indiana USA; ^2^ 32447 Department of Biology Franklin College Franklin Indiana USA

**Keywords:** Canada goose, elevated nesting, rooftop, temperate breeding, urban

## Abstract

The Canada goose (*Branta canadensis*) population has radically changed over the past 60 years—from once being extirpated in the state of Indiana to the current level of approximately 113,000. High urban densities have resulted in persistent human–wildlife conflicts and novel interactions between geese and their physical environment. Canada geese typically choose nest sites that are on the ground or slightly elevated sites such as muskrat lodge, but we report observations of Canada geese nesting on rooftops 2.6–12.2 m above ground level in central Indiana. These observations suggest that alternative, unpredicted nesting sites are being chosen over more traditional sites, in a likely attempt to reduce risks of disturbance and predation. This atypical nest‐site selection may pose new management challenges, but further research is needed.

## Graphical abstract

High urban densities of Canada geese have resulted in novel interactions between geese and their physical environment. It is understood that Canada geese choose nest sites that are on the ground or slightly elevated sites such as muskrat mounds. We report our observations of Canada geese nesting on rooftops ranging from 2.6–12.2 m above ground level in urban habitats in central Indiana.

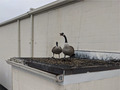

## 1 INTRODUCTION

Canada goose (*Branta canadensis*) populations in rural and urban environments have been increasing since the implementation of the Migratory Bird Treaty Act in 1918 (Baldassarre, 2014). The number of temperate breeding Canada geese in the Mississippi Flyway is estimated at 1.4 million (Luukkonen & Leafloor, 2017), with Indiana having an estimated 113,000. Existing studies of Canada goose life history describe archetypal nests—made from plant materials, down, and other body feathers (Baldassarre, 2014)—as located on the ground, at or near water, with open, unobstructed areas surrounding the nest. High abundances of Canada geese are especially apparent in urban environments, where conditions are often suitable for populations to thrive (i.e., low predation pressures, reduced hunting pressure, and ample, year‐round resources) (Balkcom, 2010; Fox, 2019).

Population increases have facilitated the encroachment of temperate‐breeding Canada geese into urban (developed) areas, where they are now considered a nuisance by many (Fox, 2019). This nuisance status is due to their defensive and aggressive nature during nesting and brood‐rearing as well as their rate of defecation. Numerous studies describe management techniques to mitigate human–goose conflicts such as the hunting of translocated geese, harassment of nuisance geese, and construction of barriers to exclude nuisance geese (Castelli & Sleggs, 2000; Holevinski et al., 2006; Mills & Combs, 2018; Smith et al., 1999). Others have explored human perspectives of nuisance geese and alternative management strategies; finding that property damage and perceived risks of Canada geese are likely indicators to support lethal or nonlethal management techniques (Coluccy et al., 2001).

Prior to the extensive recovery of Canada goose populations, wildlife managers used culverts and raised platforms to aid in nesting and recruitment (Mackey et al., 1988). These artificial nest structures were predicated on a well‐documented understanding that elevated nesting (e.g., raised platforms) minimizes nest predation risk (Anderson et al., 2015).

This phenomenon is observed in natural nesting behaviors as well. In the goose family, several species have been reported nesting on cliffs and steep slopes, including Richardson's cackling goose (*Branta hutchinsii*), barnacle goose (*Branta leucopsis*), pink‐footed goose (*Anser brachyrhynchus*), and Canada geese. Canada geese have also been reported to nest in trees and abandoned raptor nests (Lebeda & Ratti, 1983; Mackey et al., 1988; Nelson, 1953; Norment et al., 1999). Brief mention of Canada geese using elevated, man‐made structures such as bridges, pilings, and city buildings can be found in Campbell et al. (1990), but with little detail. From a broader behavioral perspective, rooftops have been documented as resting locations for Canada geese in the autumns and winters (Dorak et al., 2017). Rooftops provide low predation pressures and warmer microclimates during these wintering months, which may explain Canada goose affinity to rooftops during this time (Dorak et al., 2017).

We report here the first detailed accounts of Canada geese using rooftops as nesting locations. Additionally, we describe nest materials, nest success, and clutch sizes of rooftop‐nesting Canada geese in central Indiana. We suggest that this type of nesting behavior may provide additional challenges to managers and may produce additional nuisance behaviors associated with this species.

## 2 OBSERVATIONS

From March to July 2021, we monitored Canada goose nests in the Indianapolis Metropolitan Area (Indiana, USA). During our routine observations across three study areas, we found 5 nests on elevated rooftops. Nests were monitored on a weekly basis by capturing band information from the adults present as well as number of eggs observed. Fates for nests were assigned after hatching through observations of egg membranes and goslings present.

**FIGURE 1:**
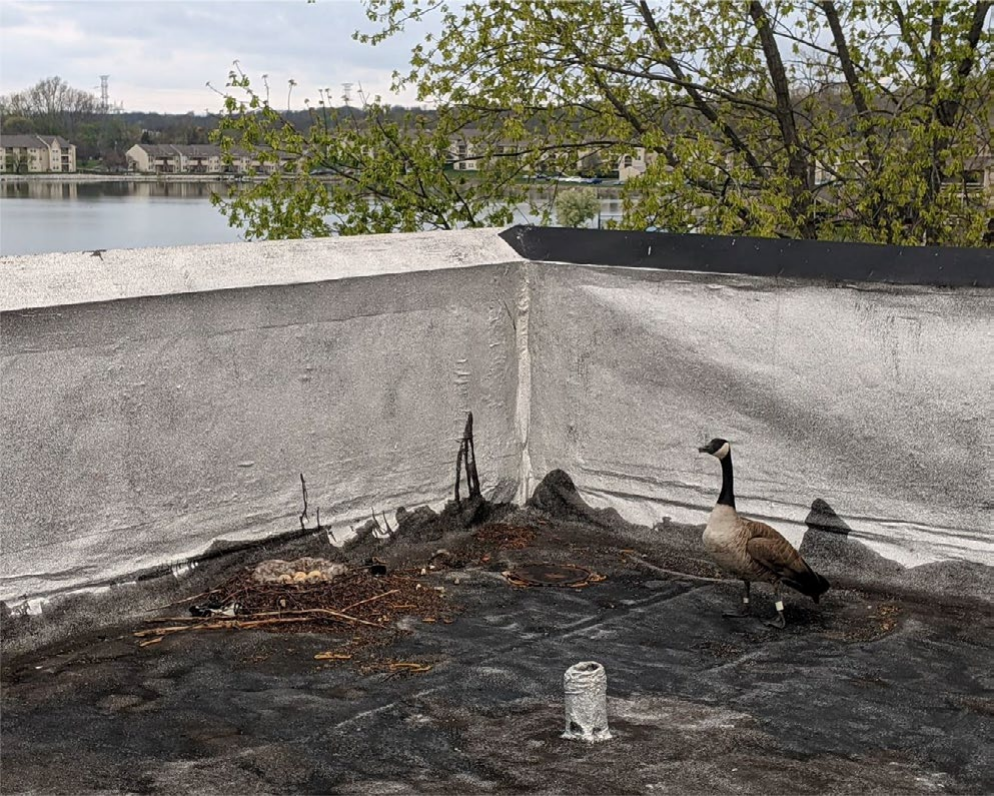
Rooftop nest (left) of a Canada goose pair (*Branta canadensis*) composed of leaf litter. Note that the entire rooftop is surrounded by a roughly 1.2 m high barrier

**FIGURE 2:**
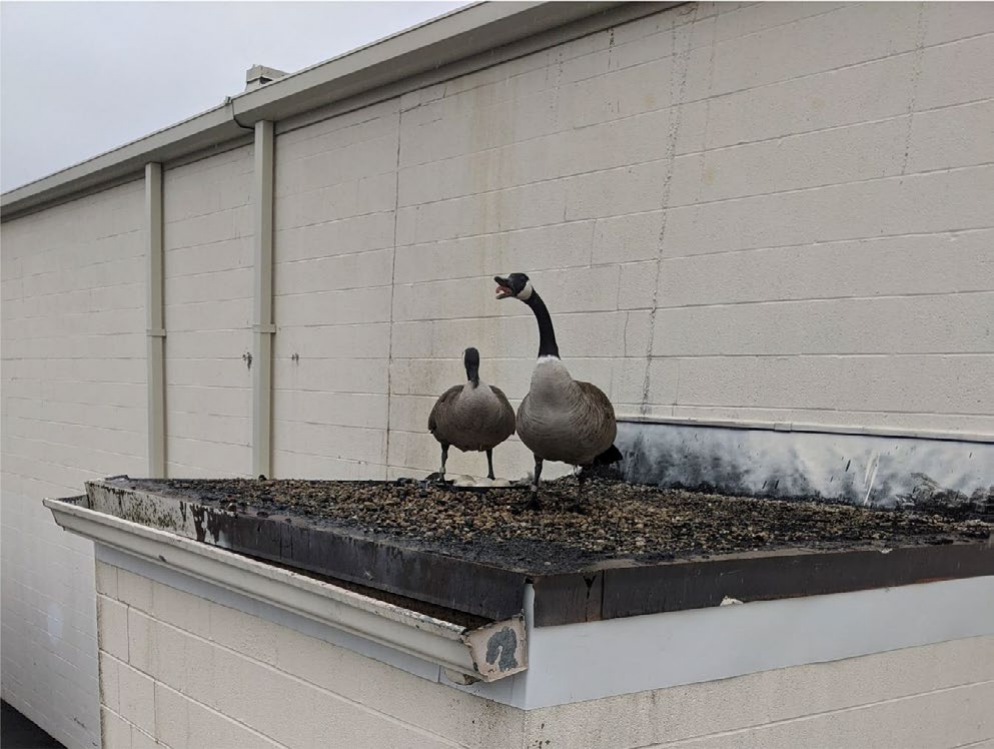
Nesting pair of Canada geese (*Branta canadensis*) defending an elevated nest. Note that the nest is composed of atypical materials of rock and a rubber automotive belt with little to no down/body feathers

**FIGURE 3:**
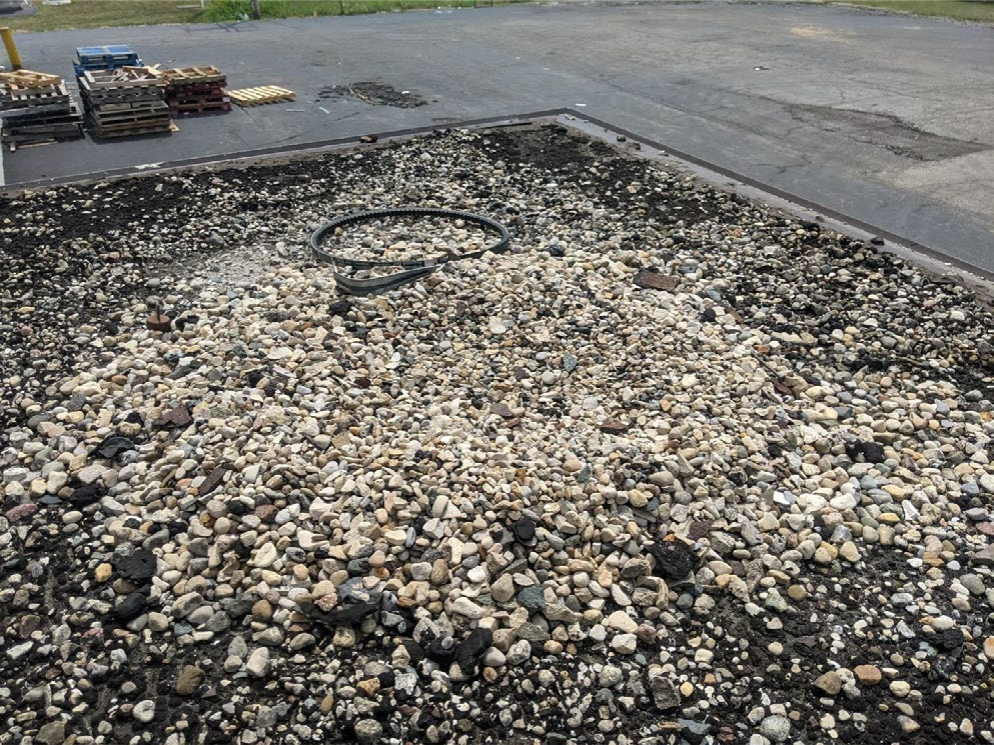
Rooftop Canada goose (*Branta canadensis*) nest post‐hatch. Note the automotive belt that was used as a “liner” in Figure 2 was moved and instead a shallow depression in gravel was used as the nest for the second time in the 2021 season

**FIGURE 4:**
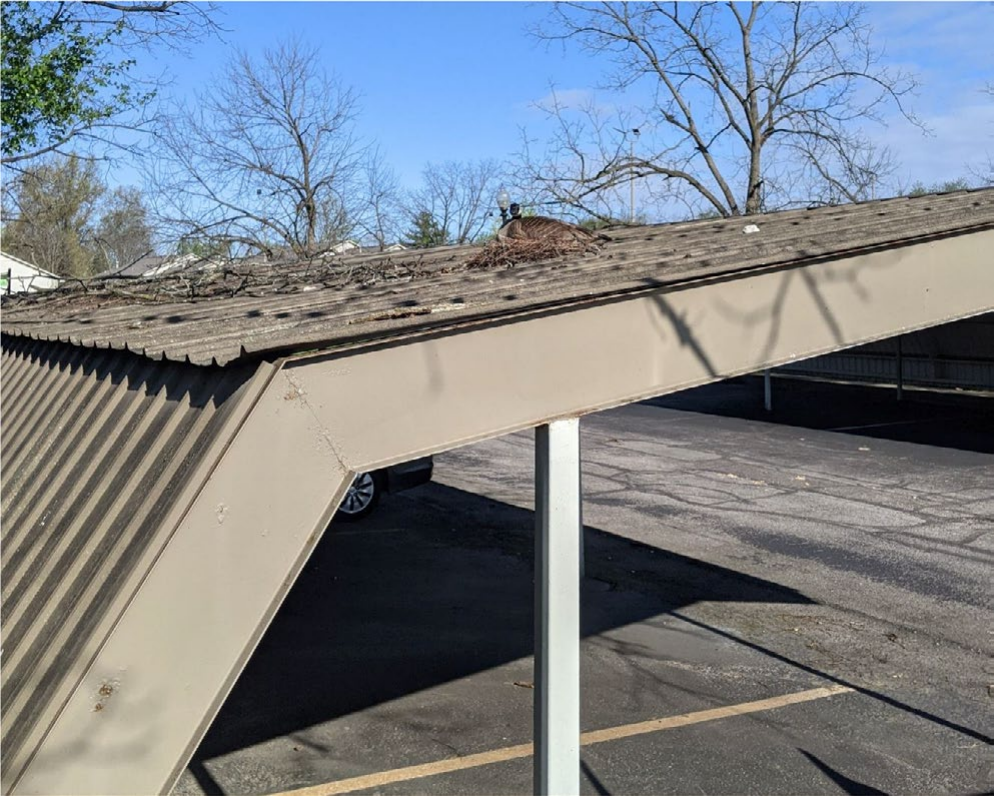
Nesting female Canada goose (*Branta canadensis*) incubating an elevated nest located on top of a carport roof in Southport, IN. Nest was composed of traditional nesting material, mainly leaf litter and twigs

**FIGURE 5:**
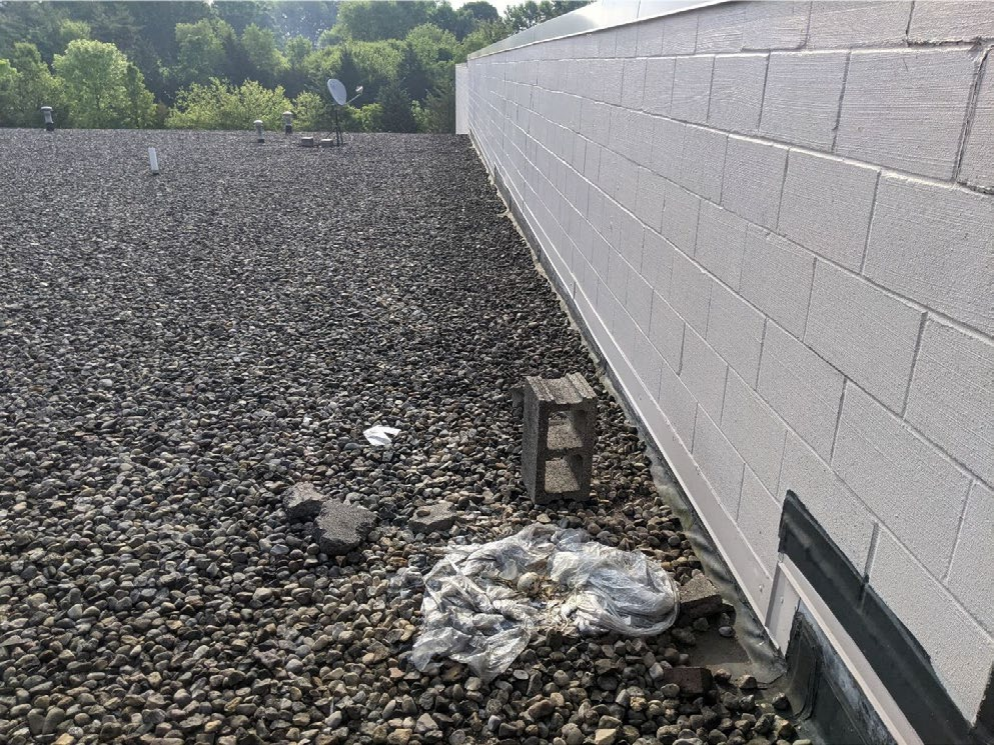
Canada goose (*Branta canadensis*) nest with atypical building materials (plastic sheet) with no down or body feathers present. Found in Southport, IN, post‐hatch

The first nest (Figure 1) was discovered on 20 April 2021 in Speedway, IN, on a hotel building that had a 1.2‐meter tall safety wall around the perimeter of the roof. The second nest (Figure 2) was discovered on 27 March 2021 in Greenfield, IN. This site was used twice in the 2021 field season by two different females. As such, we treated this as our third observation of the focal behavior (Figure 3). The females of this nesting site were differentiated due to their unique tarsal bands. The fourth nest (Figure 4) was discovered on 15 April 2021 in Southport, IN. The fifth nest (Figure 5) was discovered on 25 May 2021 in Southport, IN. Nests varied in distance from ground and distance to nearest body of water (Table 1).

**TABLE 1:** Characteristics of each Canada goose (*Branta canadensis*) nest found on rooftops

Nest	Building type	Distance from ground level (m)	Distance from nearest body of water (m)	Number of eggs observed	Number of eggs hatched	Total brood size	Nesting materials
1	Hotel	12.2	21.0	5	5	5	Leaf litter, few twigs, down/body feathers
2	Shopping Plaza	5.0	96.0	3	3	3	Loose gravel, automotive belt, little to no down
3	Shopping Plaza	5.0	96.0	4	4	4	Loose gravel, little to no down
4	Apartment Complex Carport	2.6	37.0	4	4	4	Leaf litter, twigs, down/body feathers
5	Shopping Plaza	6.0	90.9	4	3	3	Loose gravel, thin plastic sheet, little to no down

Note

Nests were discovered between March and May of 2021 during routine nest surveys of various urban sites in central Indiana, USA.

Canada geese that nested on rooftops produced fewer eggs that were more successful than geese that nested on the ground. The number of eggs per nest varied, with a significantly lower mean clutch size in elevated nests (4.00 ± 0.71) relative to non‐elevated nests (5.01 ± 1.37) in our study area (*t* = 2.82, df = 6.7, *p *= .0269). However, there was no evidence of gosling mortality observed in any of the above‐described nests. Goslings from nests 1 and 5 were rescued by researchers during routine nest checks of the field sites, indicating that rooftop nests may be potential traps for Canada goose goslings. Nevertheless, rooftop nests were 100% successful in hatching (Table 1), compared to 59.9% of nests found at ground level at our field sites (DJS unpublished data). While ground nests were typically made from plant material, feathers, and occasionally litter, rooftop nests were less likely to include feathers, and plant material, in general (Table 1).

## 3 DISCUSSION

With their growing populations, Canada geese in urban areas are increasingly viewed as a nuisance species due to their defecation, defensive behaviors, and property damage. While rooftop nesting has been documented in several avian species such as gulls (Soldatini et al., 2008), killdeer (Ankney & Hopkins, 1985), terns (Forys & Borboen‐Abrams, 2006; Warraich et al., 2012), and nighthawks (Mays et al., 2019; Newberry & Swanson, 2018), such behavior in Canada geese has not yet been well‐characterized.

This novel behavior could pose additional challenges in the form of new human–goose conflicts and could present additional need for management intervention. For example, rooftop nesting geese may be considered a nuisance to homeowners and property managers as they are defensive and territorial during the nesting period. Interventions such as nest destructions or aiding goslings during nest departure, particularly if the rooftop has a barrier wall, will be necessary to mitigate human–goose conflicts.

We hypothesize that Canada geese may be selecting elevated nesting sites in urban areas to avoid predation and/or disturbance from ground predators, which would include typical mammalian predators (e.g., coyotes, foxes, and raccoons) and humans. This hypothesis is supported by data showing that elevated nests are more successful than ground nests in Canada geese (Krohn & Bizeau, 1980). Canada geese nesting on rooftops may have unsuccessful clutches from extreme heat, low gosling survival from nest departure, or lack of vital resources that are not found on roofs. Other studies indicate that structures and obstacles near the nest such as predator‐proof fencing may pose a threat to brood survivability (Howerter et al., 1996; Trottier et al., 1994). Additionally, a number of studies suggest that extreme heat has a negative effect on egg viability in various avian species (Beissinger et al., 2005; Saino et al., 2004; Stoleson & Beissinger, 1999), though this did not appear to have any effect on the viability of Canada geese eggs in our study areas.

Our documentation here provides additional evidence to the myriad of ways wildlife species are interacting with novel human environments. Additional research is warranted to assess the frequency of elevated nesting across broader spatial scales and the fate of rooftop nests in metropolitan areas. Further understanding of this behavior could become quite useful to our future management of this important species.

## CONFLICT OF INTEREST

The authors declare that they have no conflicts of interest.

## AUTHOR CONTRIBUTIONS

**David J. Shearer:** Conceptualization (equal); Writing – original draft (lead). **Timothy C. Carter:** Funding acquisition (lead); Supervision (equal); Writing – review & editing (equal). **Benjamin J. O'Neal:** Conceptualization (equal); Methodology (equal); Writing – review & editing (equal).

## Data Availability

All data are present in the publication.
